# Multiple Pilomatricoma in a Middle-Aged Woman

**DOI:** 10.7759/cureus.18475

**Published:** 2021-10-04

**Authors:** Shoaib Muhammad, Amman Yousaf, Aribah Atiq, Ahmed Munir, Syed I Alam

**Affiliations:** 1 Urology, Ghulab Devi Hospital, Al-Aleem Medical College, Lahore, PAK; 2 Internal Medicine, McLaren Hospital, Flint, USA; 3 Pathology, Chughtai Laboratory, Lahore, PAK; 4 Orthopaedics and Rehabilitation, Hamad Medical Corporation, Doha, QAT; 5 Radiology, Hamad Medical Corporation, Doha, QAT

**Keywords:** neurofibromas, skin lesions, pilomatricoma, skin tumor, soft tissue swelling

## Abstract

Pilomatricomas are rare skin tumors related to hair follicles. They typically present in children, and the most common locations are head and neck. Pilomatricomas are usually painless; however, they can cause cosmetic problems. Treatment is decided on an individual basis and involves local excision. However, caution should be taken if the lesion is malignant, as resection with safe margins (0.5-1.0 cm) is determinant of the overall outcome. Radiotherapy is indicated in cases of residual tumor tissue or recurrence. The role of chemotherapy in pilomatricomas is still undetermined. We report a 55-year-old female with multiple lumps on her shoulder and back. The sonographic features of these lesions were typically consistent with pilomatricomas. The patient decided to opt for excision due to cosmetic reasons and the histopathology features were suggestive of pilomatricoma.

## Introduction

A pilomatricoma, also known as “calcifying epithelioma of Malherbe,” is a rare skin tumor. Malherbe and Chenantias first described this tumor in 1880 [1]. Pilomatricoma is derived from hair matrix cells and typically grows in the hair follicles [2]. It usually presents as a nodule or papule in the head and neck regions. Children and adults in their 20s are more likely to be affected by pilomatricomas. The lesions are generally painless; however, patients opt for excision due to cosmetic reasons. Incidence of multiple pilomatricomas is quite rare, and only a few cases are reported in the literature. We present a 55-year-old patient who presented with numerous lesions and was diagnosed with multiple pilomatricoma radiologically, which were later confirmed by excisional biopsy and histopathology.

## Case presentation

A 55-year-old female patient with no significant past medical history presented to our hospital with a painless swelling behind the right shoulder and upper back for two years. The swellings were stable in size; however, the patient documented recurrent infection of the lesions for which she took antibiotics. On examination, there were multiple subcutaneous bumps behind the right shoulder and upper back. The swellings were solid, well-defined, with no punctum or sinus, and the skin was intact with no signs of infection.

**Figure 1:**
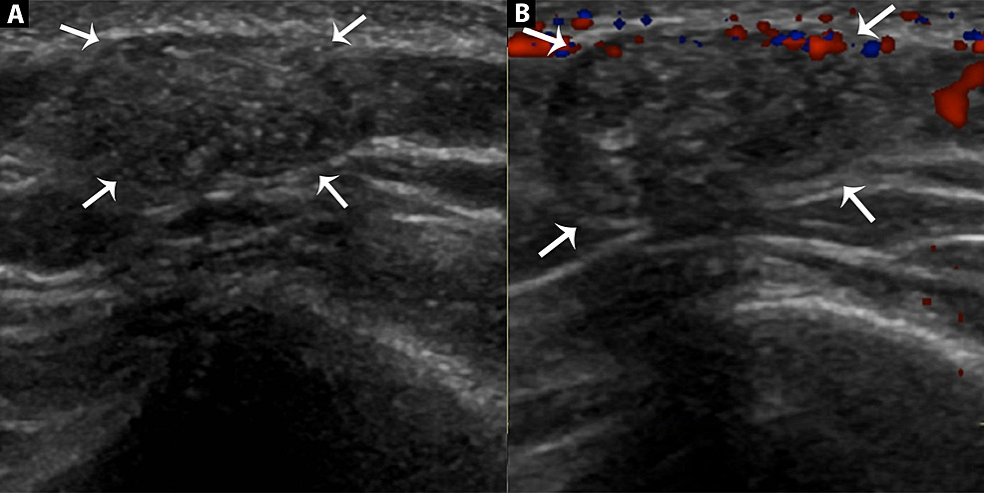
Ultrasound of the right shoulder (A) Greyscale ultrasound of shoulder showing, an oval-shaped, well-defined isoechoic lesion, having hypoechoic margins (white arrows) and internal calcifications mainly in the peripheral regions. It is associated with focal thinning of subcutaneous fat plains. (B) Doppler ultrasound showing the absence of vascularity within the lesion, consistent with the benign nature of the swelling.

Ultrasound soft tissue was done for further evaluation, which demonstrated a benign lesion in the subcutaneous region of the right posterior shoulder with a few calcifications and peripheral halo sign typically consistent with pilomatricoma (Figures 1A, 1B).

**Figure 2:**
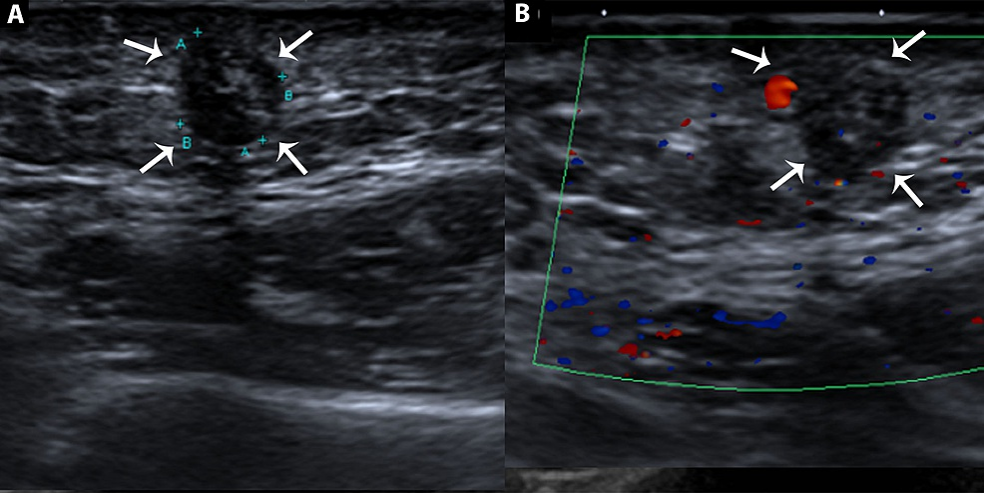
Ultrasound of cheek (A) Greyscale ultrasound of cheek showing an oval-shaped, well-defined isoechoic lesion, having hypoechoic margins (peripheral halo) and internal calcifications mainly in the peripheral regions (white arrows). It is associated with focal thinning of subcutaneous fat plains. (B) Doppler ultrasound of the cheek showing the absence of vascularity in the lesion (white arrows), consistent with the benign nature of the swelling. (A+ to A+ dimension of the lesion= 3 cm and B+ to B+ dimension of the lesion measures 2 cm).

Lesions with similar sonographic features were also seen in the lower cervical region and the left cheek (Figures 2A, 2B).

**Figure 3:**
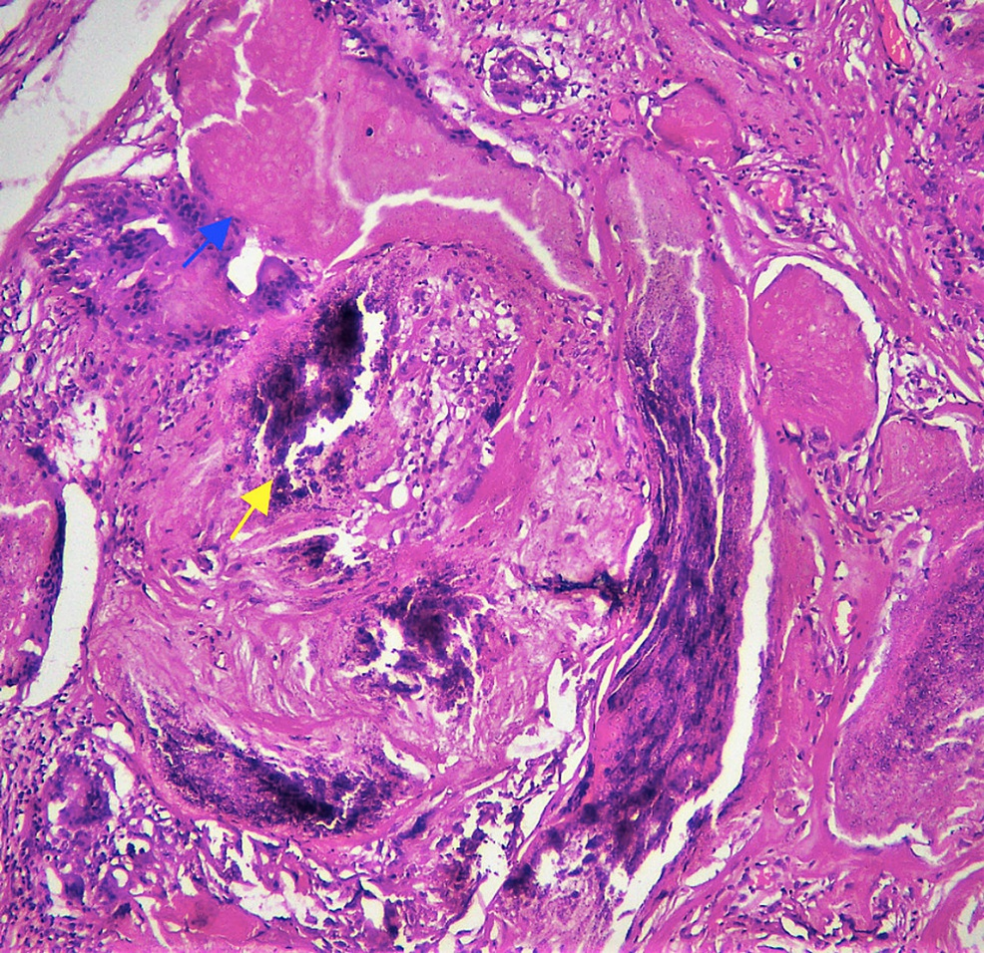
Histopathology section of the excised lesion It shows an encapsulated lesion with ghost cells (blue arrow) and areas of calcification (yellow arrow).

The patient was counseled about the benign nature of the swelling; however, she opted for surgical excision due to cosmetic reasons. Histopathology confirmed the presence of encapsulated and circumscribed lesions having basaloid and ghost cells (Figure 3).

On follow-up visits, after three and six months, there was no reoccurrence of the lesions and the patient is healthy to date.

## Discussion

Pilomatricomas are rare skin tumors and present as superficial firm nodules. They are typically painless and only rarely show overlying skin changes. Typical presentation includes a firm, reddish or bluish, moveable subcutaneous nodule usually having a lobulated surface similar to the exhibition in our patient [3,4]. Due to highly non-specific features, it is possible to confuse pilomatricoma with various soft tissue swellings like skin cancers, dermoid and epidermal cysts. Pilomatricomas usually present a solitary lesion, and only a few cases of multiple pilomatricomas are reported as in our patient [5].

Epidemiological studies suggest a bimodal pattern of incidence [3]. The first group includes young patients, while the second group contains patients in their 50s. Polimatricomas have female predominance and commonly involve the head and neck [4,6]. However, our patient showed swelling in the upper shoulder and back regions.

Multiple cellular and molecular pathways are thought to play a role in the development of pilomatricomas. Out of these, CTNNB1/Wnt/β-Catenin signaling pathway is the most widely demonstrated in the pathogenesis of pilomatricomas [7]. Additionally, a recent study through whole-exome sequencing (WES) and mutational sequencing (MS) analysis demonstrated a distinct fibroblast growth factor receptor-4 (FGFR4) mutation in pilomatricoma development. However, further studies at a relatively large scale are needed to find genetics role in the pathogenesis.

Histologically, pilomatricomas are follicular neoplasms, with well-defined circumscribed cystic structures having soft borders [8]. Pilomatricomas contain mainly three types of cells. The matrical cells having a close resemblance with hair follicles are basophilic. The other kinds of cells include necrotic anucleic “shadow cells or ghost cells,” like in our case and the third type is intermediate cells with properties overlapping between the two cell groups. In rare instances, the basophilic cells population may also contain melanin pigment [9]. Other less common features include metaplastic ossification, granulomatous reactions related to foreign-body, calcification, and necrosis. It is essential to note the depth of invasion of the lesion, presence of mitotic figures, necrosis, and the presence of perineural and vascular invasion. These features, if present, point towards the lesion being malignant.

Various radiological modalities have been implicated in diagnosing pilomatricoma. Out of these, ultrasound is the primary for its easy accessibility. Pilomatricomas appear as solid isoechoic tumors on ultrasound and a clear hypoechoic outline. These tumors are typically completely calcifying; however, some tumors show partial calcification. A computed tomography (CT) scan yields better images with more details of calcifications. On MRI, pilomatricoma appears as homogenous (66.7%) or inhomogeneous (33.3%), hypointense lesions on T1-weighted images with ring-like enhancement on post-contrast images. A few of the features of fat-suppressed T2-weighted images include reticular hyperintensity, peri-tumoral fat stranding, and circular target sign [10].

Treatment is usually local excision for the sake of cosmetic reasons in case of benign lesions. However, caution should be taken if the lesion is malignant, as resection with safe margins (0.5-1.0 cm) is crucial to the overall outcome [11]. Other modalities like radiotherapy are indicated if there is residual tissue left or if there is reoccurrence. However, the use of chemotherapy is still not established [9].

## Conclusions

Pilomatricomas are rare skin tumors related to hair follicles, mostly present in children. These tumors are primarily found in the head and neck and are mostly asymptomatic and have a reddish or bluish appearance. Typically, pilomatricomas are 5-10 mm in size, regular or irregular in shape, and can be tender on palpation. Ultrasound can help in the diagnosis and typically presents as an isoechoic lesion with peripheral halo sign. Management is usually conservative; however, some patients opt for excision due to cosmetic regions. Histopathology shows encapsulated and circumscribed lesions having basaloid and ghost cells with areas of calcification and keratinization. More research is needed to further understand the pathophysiology of pilomatricomas.
